# Associations of *LIG4* and *HSPB1* Genetic Polymorphisms with Risk of Radiation-Induced Lung Injury in Lung Cancer Patients Treated with Radiotherapy

**DOI:** 10.1155/2015/860373

**Published:** 2015-02-25

**Authors:** Feng Xu, Ji-Chang Han, Ya-Jun Zhang, Yi-Jie Zhang, Xiao-Chun Liu, Guan-Bin Qi, Dan Liu, Yan-Zhi Chen, Yu-Xia Zhao, Lu Bai

**Affiliations:** Department of Respiration, Huaihe Hospital of Henan University, Kaifeng 475000, China

## Abstract

*Objective.* This study aims to explore the correlations of genetic polymorphisms in *LIG4* and *HSPB1* genes with the radiation-induced lung injury (RILI), especially radiation pneumonitis (RP), in lung cancer patients. *Methods.* A total of 160 lung cancer patients, who were diagnosed with inoperable lung cancer and received radiotherapy, were included in the present study from September 2009 to December 2011. TaqMan Real-Time PCR (RT-PCR) was used to verify the SNPs of *LIG4* and *HSPB1* genes. Chi-square criterion was used to compare the differences in demographic characteristics, exposure to risk factors, and SNPs genotypes. Crude odds ratios (ORs) with 95% confidence intervals (95% CI) were calculated by logistic regression analysis. All statistical analyses were conducted in SPSS 18.0. *Results.* A total of 32 (20.0%) lung cancer patients had RP after receiving radiotherapy. Of the 32 cases, 4 cases were of grade 2, 24 cases were of grade 3, and 4 cases were of grade 4. However, our results indicated that the general condition and treatment of all patients had no significant difference with RP risk (*P* > 0.05). Meanwhile, our results revealed that there was no significant association between the frequencies of *LIG4 rs1805388* and *HSPB1 rs2868371* genotype distribution and the risk of RP (*P* > 0.05). *Conclusion.* In conclusion, we demonstrated that the genetic polymorphisms in *LIG4 rs1805388* and *HSPB1 rs2868371* were not obviously correlated with the risk of RP and RILI of lung cancer.

## 1. Introduction

Lung cancer is the most common cause of cancer death all over the world, with a 5-year overall death rate of 10%~15% in an increasing trend [1]. Currently, the main treatments of lung cancer include chemotherapy, surgery, and radiotherapy, among which most of lung cancer patients were treated with radiotherapy which can easily lead to radiation-induced lung injury (RILI) [2]. RILI, one of the most common complications during or after the treatment process of lung cancer, is one of the main limiting factors of radiotherapy and chemotherapy for malignant tumors [3]. In addition, the RILI presented with two main stages in the progression of damage, including radiation pneumonitis (RP) of the early stage and radiation fibrosis of the advanced stage [4]. About 10%~20% of lung cancer patients receiving radiotherapy commonly present with the symptoms of RILI including congestion, dry cough, low fever, a sensation of chest expansion, and pleuritic chest pain [5]. It is estimated that the incidence of moderate to severe RILI varies from 10% to 20%, and RILI has been threatening people's normal life and living quality seriously [6]. Based on the clinical observations, there are huge differences in radiation-induced injury of normal tissues among the patients with similar age, nutritional status, and radiophysics factors, so it is possible that the individual radiobiology may affect the degree of RILI [4, 7]. Additionally, numerous studies have also shown that the single nucleotide polymorphisms (SNPs) of various genes may be connected with the individual radiobiology [8, 9].

*Ligase IV (LIG4)*, a human gene located on chromosome 13q33 to q34 containing 5 exons and 4 introns to encode the DNA Ligase IV, which could be acted as an ATP-dependent DNA ligase directly mediating DNA-strand joining, is central to nonhomologous end joining (NHEJ) by forming a protein complex with XRCC4 [10–12]. The complex catalyzes the final ligation of NHEJ pathway of DNA double-strand break (DSB) repair, ability of which has a great influence on radiosensitivity of tumor cells [13, 14]. As have been confirmed, the genetic polymorphisms of NHEJ repair genes can modulate the RILI risk in non-small cell lung cancer (NSCLC) patients under radiotherapy treatment; for example, the loss or hypomorphic mutations of* LIG4* can contribute to increased radiosensitivity and cause LIG4 syndrome, a disease with many characters including pronounced radiosensitivity and malignancy [15, 16]. Therefore, the SNP of* LIG4* may be associated with the increased risk of lung cancer and may be served as a very important marker in relation to the susceptibility to clinical radiosensitivity and RILI [15, 17]. The heat shock protein beta 1 (HSPB1), also known as heat shock protein 27 (HSP27), is an important member in the family of the small heat shock protein [18]. HSPB1 is normally expressed at low levels and can be activated by sundry environmental or physiological stresses including heat shock, cytotoxic or apoptotic stimuli, and oxidative stress [18, 19]. Being a molecular chaperone independent of adenosine triphosphate, HSPB1 can play a simulative role in repairing or degrading damaged proteins emerging in cells exposed to stress [20]. What is more, HSPB1 enhanced the antioxidant defense capacity of cells by increasing glutathione cell content [21], and its chaperone activity relieved the toxic effects of oxidized proteins [22]. That may matter a lot for cells to respond to irradiation because reactive oxygen species play a determinant role in inducing apoptosis [23]. In addition, HSPB1 is in charge of neutrophil apoptosis and is related with F-actin, an actin maintaining cytoskeleton structure and avoiding its disaggregation [22]. As HSPB1 is encoded by the* heat shock protein beta-1* gene (*HSPB1*), mapped to chromosome 7 at q11.23 and containing 3 exons and 2 introns, the expression level of HSPB1 protein may be associated with the* HSPB1* genotype, which was connected with the pathogenesis of various diseases. The present study is conducted to investigate whether the genetic polymorphisms of* LIG4* and* HSPB1* are involved in the increased risk of RILI among patients with lung cancer after radiotherapy.

## 2. Materials and Methods

### 2.1. Ethics Statement

The study design was reviewed and approved by the Institutional Review Board of the Huaihe Hospital of Henan University. All procedures in this study were in compliance with the Declaration of Helsinki. The informed written consent was obtained from all patients prior to study.

### 2.2. Patient Eligibility

From September 2009 to December 2011, a total of 160 medically inoperable patients with lung cancer in Henan province were enrolled in this present study. All the patients had histologically and pathologically confirmed diagnosis and received radiation therapy. All eligible patients had a Karnofsky performance status (KPS) >60 scores and a life expectancy >6 months. Patients were excluded for any of the following conditions: (1) having previous and coexistent thoracic radiotherapy; (2) having pulmonary surgery; (3) having severe chronic bronchitis, emphysema, and pulmonary heart disease; (4) with FVC < 60% predict; (5) having suffered from other severe diseases, such as myocardial infarction within 6 months.

### 2.3. Radiation Therapy

Before the spiral CT scans (Toshiba, Japan), patients were immobilized using vacuum cushions. Spiral CT scanning in the lesion area was performed with 5 mm slice thickness and transmitted the images to the three-dimensional treatment planning system (Pinnacle3, ADAC) to delineate the target volume and vital organs. The gross tumor volume (GTV) was consisting of the primary tumor and mediastinal lymph nodes with short-axis diameter over 1 cm. The planning target volume (PTV) was expanded on the basis of the CTV by 1.0–1.5 cm. All patients in this study were treated with coplanar or noncoplanar for conformal irradiation and the exposure doses of spinal or other vital tissues and organs should be controlled. The therapy plan was estimated using dose volume histograms and isodose curves. The three-dimensional conformal radiation (3D-CRD) therapy was performed using the linear accelerator (SIMENS, PRJMUS). Ninety percent of isodose curves were regarded as the prescription dose line. A total dose of 50–60 Gy was applied and all radiation fields were treated once per day, six times per week.

### 2.4. Chemotherapy

A total of 30 lung cancer patients received radiation alone in this study, while 130 lung cancer patients received chemotherapy. Among the 130 lung cancer patients, 20 patients received concurrent chemoradiotherapy (CCRT) and were treated with platinum-based two-drug combination. A course of treatment was 21 days and 2 cycles of radiation were completed within the period of chemotherapy. Other 110 patients received sequential chemoradiotherapy (SCRT) and were also treated with platinum-based two-drug combination.

### 2.5. Pulmonary Lesions Assessment

The evaluation of lung pulmonary lesions was based on the assessment of acute radiation-induced pulmonary. According to the National Cancer Institute's Common Toxicity Criteria (NCI CTC3.0) [24], five separate grades were distinguished: (1) grade 0, having no obvious change in symptoms and signs compared with pretreatment; (2) grade 1, having no obvious change in respiratory symptoms, only X-ray film with inflammation; (3) grade 2, having persistent cough and requiring narcotic antitussive and having no influence on the normal life; (4) grade 3, having severe cough and being poorly controlled by narcotic antitussive, having serious effect on human's normal life, and requiring intermittent oxygen therapy or applying corticosteroids; (5) grade 4, having severe respiratory insufficiency, requiring continuous oxygen or ventilator support, and being actually or potentially life-threatening; (6) grade 5, resulting in death from severe radiation pneumonitis. The following situations should be excluded when diagnosing the radiation pneumonitis (RP): (1) pulmonary infection; (2) disease progression in pulmonary; (3) related clinical symptomatic grading which should increase at least one grade compared with pretreatment. Grade of radiation pneumonitis ≥2 was regarded as the endpoint grade.

### 2.6. DNA Extraction and Genotyping Method

All the eligible patients provided 2 mL peripheral blood collected by sodium citrate and then stored in a refrigerator with the temperature −20°C or −80°C. Phenol-chloroform method was used to extract DNA products. After DNA extraction, OD value was detected to confirm the original concentration of DNA products and then diluted to 30–100 ng/*μ*L to reserve. Real-Time PCR TaqMan Assay was performed to genotype the SNP rs1805388 of* LIG4* gene and rs2868371 of* HSPB1* gene.

### 2.7. Statistical Analysis

All statistical analyses were carried out using SPSS 18.0 software and a standard two-tailed test with *P* < 0.05 was adopted for significance. *χ* ^2^ test was performed to compare the demographic characteristics, exposure rates of risk factors, and the differences between the distribution of SNPs. Crude odds ratios (ORs) with 95% confidence intervals (95% CI) were calculated by logistic regression analysis.

## 3. Results

### 3.1. Baseline Characteristics

Baseline clinical characteristics of all patients are shown in Table 1. The present study included 160 patients with lung cancer (112 NSCLC and 39 SCLC) with a median age of 59.0 years (range, 25–75 years), of whom 78.8% (126/34) were males. Among these 160 patients, 15 had stages I~II and 145 had stages III~IV. Regarding smoking status, 75.0% (*n* = 120) of the patients were smokers. Of these 160 patients, nearly 81.2% (*n* = 130) of them received platinum-based two-drug combination chemotherapy including 20 patients treated by CCRT, who received two cycles of chemotherapy concurrently with RT, and 110 patients treated by SCRT; in addition, 30 (18.8%) patients received RT alone.

### 3.2. Clinical Characteristic and Radiation Pneumonitis

After radiotherapy, there were occurrences 32 (20.0%) of grade ≥2 radiation pneumonitis (RP) in our population (grade 2, grade 3, and grade 4 were observed in 4, 24, and 4, resp.) (Table 2). The incidence of RP consisted of 24 men and 8 women (Table 2). There were no significant differences between patients who developed grade ≥2 RP in univariate analyses with regard to count data including gender, smoking status, histology, clinical stage, and type of treatment (Table 2) and measurement data including age, V20 value, and gross tumor volume (GTV) (Table 3).

### 3.3. LIG4 and HSPB1 Genetic Polymorphisms and the Risk of Radiation Pneumonitis

Table 4 listed the associations of LIG4 (rs1805388) and HSPB1 (rs2868371) genotypes with the risk of grade ≥2 RP in univariate analyses. In general, the incidences of grade ≥2 RP observed in patients of the rs1805388 CC and rs2868371 GG genotypes were 81.2% (26/32) and 42.43.8% (14/32), which were not statistically significantly different from those of the rs1805388 CT/TT (18.8%, *P* = 0.140) and rs2868371 GC/CC genotypes (56.2%, *P* = 0.628). Genotype frequencies were all in HWE (all *P* > 0.05).

## 4. Discussion

Nowadays, lung cancer has been considered to be an aggressive malignancy with short overall survival, which was the leading cause of cancer death all around the world [25]. In the past few decades, surgical treatments, radiotherapy, and multiple cycles of chemotherapy were the main therapies in the treatment of lung cancer [26–28]. However, along with the wide application of radiotherapy, the radiation-induced normal tissue injury has been taken into the consideration of evaluating the efficacy of radiotherapy treatments for lung cancer patients [29, 30]. RILI has been regarded as one of the most common complications after the treatment of radiotherapy or concurrent chemoradiotherapy, which could largely restraint the efficacy of lung cancer patients and might affect the survival time and quality of life of patients with lung cancer [29, 31]. Meanwhile, the RILI could be regarded as the progression of damage, including radiation pneumonitis (RP) in the early stage and radiation fibrosis in the advanced stage [4].

In the present study, the primary objectives were to investigate the incidence of RP of lung cancer patients after the treatment of radiotherapy and to analyze the association between the* LIG4* and* HSPB1* genes polymorphisms and RILI in lung cancer patients. The key findings in this study have revealed that the genetic polymorphisms of* LIG4* (rs1805388) and* HSPB1* (rs2868371) were not evidently associated with the RILI in lung cancer patients, implying that the genetic polymorphisms of* LIG4* and* HSPB1* may not induce or inhibit the radiation-induced normal tissue injury in the lung cancer patients after the treatment of radiotherapy. To this day, previous studies have demonstrated that genetic mutations in tumor-related genes may lead to the dysfunction of DNA repair, and the abnormal expression of tumor-related genes may be implicated in the process of cell growth, proliferation, differentiation, and apoptosis, which may finally result in carcinogenesis [32, 33]. It has also been shown that various genetic variations in tumor genes were involved in the glutathione and the pathways of DNA repair, which were connected with the development and progression of lung cancer [34, 35]. Notably, the DNA double-strand break (DSB) repair pathway consisted of two main DNA repair pathways, including homologous recombination (HR) and nonhomology end joining (NHEJ), both of which mainly repaired the ionizing radiation-induced DNA damage [36, 37]. In mammalian cells, the DNA DSB repair was mainly achieved by activating the NHEJ pathway; and the repair of NHEJ pathway was achieved by DNA-dependent protein kinase (DNA-PK) and the XRCC4/Lig4 complex [38, 39]. It has been reported that the functional incapacitation of XRCC4/Lig4 complex could have negative effects on the DNA DSB repair and could result in the enhancement of radiosensibility [36, 40, 41]. It was worthwhile to note that DNA Ligase IV syndrome was considered to be a rare congenital disease, which was caused by the genetic mutations in* LIG4* and might result in the immune system of patients being extremely sensitive to ionizing radiation [16]. Furthermore, Tseng et al. have suggested that the* LIG4* genetic polymorphisms, especially rs1805388 C>T, were positively associated with the susceptibility to lung cancer, and the genetic mutations in* LIG4* may lead to a poor prognosis for patients with lung cancer [17]. A previous study performed by Yin and his colleagues has suggested that* LIG4* genetic polymorphisms (rs1805388 C>T) could increase the risk of serve RP in patients with NSCLC after receiving radiotherapy [15]. Nevertheless, we found that the* LIG4* genetic polymorphisms (rs1805388 C>T) were not significantly connected with the RILI in lung cancer patients. We presumed that the genetic polymorphisms of* LIG4* may closely relate to the ethnicity, and the frequency of genotype at the same site may differ in different ethnicity.

On the other hand, radiation may induce the occurrence of DSB, which may promote the production of cytokines and finally result in chronic oxidative stress and irreversible damage in human bodies [42]. It also has been reported that* HSPB1* gene could enhance the cells resistibility against the radiation through downregulating the inflammatory reaction; hence, the genetic variations in* HSPB1* may closely be related to the radiation damage [43]. A previous study has showed that the genetic variations in CG/GG genotypes of* HSPB1* (rs2868371) were correlated with a low risk of radiation-induced esophageal toxicity in NSCLC patients who were treated with chemoradiotherapy [22]. In addition, Pang et al. have suggested that the genetic polymorphism of* HSPB1 rs2868371* was connected with severe RP after chemoradiotherapy for NSCLC patients, especially in the patients with grade ≥3 RP [44]. In our study, we found that the* HSPB1* genetic polymorphism (rs2868371) was not evidently associated with the risk of RILI and RP, which was inconsistent with previous study [22]. We suspected that the different pathological types in lung cancer may result in the differences in genotype distribution frequency of* HSPB1*, and the frequency of genotype at the same site (rs2868371) may differ in different ethnicity. Besides, sample size was considered to be a very crucial parameter when investigating or exploring the genetic effect of any gene polymorphism, and the findings in our study may be limited by the small sample size; so we need further study to better understand the association between the* HSPB1* genetic polymorphism and the RILI.

In summary, we found no evidence of correlation between the genetic polymorphisms of* LIG4* and* HSPB1* and the development and progression of RILI, and the polymorphism in* LIG4* and* HSPB1* genes may not be potential biomarkers and was unlikely to be of substantial significance regarding RILI risk.

## Figures and Tables

**Table 1:** Patient demographics and baseline disease characteristics.

	Patients (*n* = 160)	Ratio (%)
Age (years)	59.0 (25–75)	
Gender		
Male	126	78.8
Female	34	21.2
Smoking status		
Smoker	120	75.0
Nonsmoker	40	25.0
Histological findings		
Squamous cell carcinoma	50	31.2
Adenocarcinoma	40	25.0
Others	15	9.4
Small cell carcinoma	55	34.4
Stages of lung cancer		
I	5	3.1
II	10	6.2
II	110	68.8
IV	35	21.9
Chemotherapy		
Yes	127	79.4
No	33	20.6
Types of chemotherapy		
CCRT	30	18.8
SCRT	20	12.5
	110	68.7
GTV (cm^3^)	127.54 (13.12–780.36)	
*V*20 (lung-PTV) (%)	36 (3–75)	

CCRT: concurrent chemoradiotherapy; SCRT: sequential chemoradiotherapy; GTV: gross tumor volume; PTV = planning target volume; *V*20 = percentage volume of structure receiving dose of 20 Gy or more.

**Table 2:** Univariate analysis of count data for the risk of grade ≥2 radiation pneumonitis after radiation therapy in patients with lung cancer.

Parameter	Variable	Patients (*n* = 160)	Number of RPs	*P*
Gender	Male	126	24	0.640
	Female	34	8	

Smoking status	Yes	120	21	0.273
	No	40	11	

Histology	NSCLC	105	25	0.167
	SCLC	55	7	

Stage	I	5	0	0.292
	II	10	4	
	III	110	24	
	IV	35	4	

Treatment	RT alone	30	6	0.630
	CCRT	20	6	
	SCRT	110	20	

RP: radiation pneumonitis; NSCLC: non-small cell lung cancer; SCLC: small cell lung cancer; SCRT: sequential chemoradiotherapy; CCRT: concurrent chemoradiotherapy; RT: radiotherapy.

**Table 3:** Univariate analysis of measurement data for the risk of grade ≥2 radiation pneumonitis in patients with lung cancer after radiation therapy.

Parameter	Number of patients	Median	Range	*P*
Age (years)	160	59	25~75	160
*V*20 (%)	160	36	3~75	160
GTV (cm^3^)	160	127.54	13.12~780.36	160

GTV: gross tumor volume; *V*20 = percentage volume of structure receiving dose of 20 Gy or more.

**Table 4:** Correlations of different genotypes in *LIG4* (rs1805388) and *HSPB1* (rs2868371) genetic polymorphisms with the risk of radiation pneumonitis in patients with lung cancer after radiation therapy.

Genotype	RP (%)	Non-RP (%)	OR (95% CI)	*P*
*LIG4* rs1805388				
CC	26 (81.2)	87 (68.0)	1	—
CT	6 (18.8)	31 (24.2)	0.648 (0.244–1.722)	0.381
TT	0 (0)	10 (7.8)	NC	NC
CT+TT	6 (18.8)	41 (32.0)	0.490 (0.187–1.282)	0.140
*HSPB1* rs2868371				
GG	14 (43.8)	50 (39.1)	1	—
GC	14 (43.8)	59 (46.1)	0.848 (0.369–1.946)	0.696
CC	4 (12.4)	19 (14.8)	0.752 (0.220–2.574)	0.649
GC+CC	18 (56.2)	78 (60.9)	0.824 (0.376–1.805)	0.628

RP: radiation pneumonitis; LIG4: Ligase IV; HSPB1: heat shock protein beta 1; OR: odds ratios; 95% CI: confidence intervals; NC: negative control.
